# Resurrecting Brinley Plots for a Novel Use: Meta-Analyses of Functional Brain Imaging Data in Older Adults

**DOI:** 10.1155/2008/167078

**Published:** 2007-11-06

**Authors:** Ann M. Peiffer, Joseph A. Maldjian, Paul J. Laurienti

**Affiliations:** Department of Radiology, Wake Forest University School of Medicine, Medical Center Boulevard, PP1 - 7th Floor, Winston-Salem, NC 27157, USA

## Abstract

By plotting response times of young and older adults across a variety of tasks, Brinley spurred investigation and debate into the theory of general cognitive slowing. Though controversial, Brinley plots can assess between-task differences, the impact of increasing task demand, and the relationship between responses in two groups of subjects. Since a relationship exists between response times and the blood-oxygen level dependent (BOLD) signal of functional MRI (fMRI), Brinley's plotting method could be applied as a meta-analysis tool in fMRI studies of aging. Here, fledgling “Peiffer plots” are discussed for their potential impact on understanding general cognitive brain activity in aging. Preliminary results suggest that general cognitive slowing may be localized at the sensorimotor transformation in the precentral gyrus. Although this meta-analysis method is naturally used with imaging studies of aging, theoretically it may be applied to other study pairs (e.g., schizophrenic versus normal) or imaging datasets (e.g., PET).

## 1. INTRODUCTION

In the mid 1960s, Brinley presented a novel plotting method to consider the relationship between response times in young and older adults across a variety of tasks with varying levels of cognitive difficulty [1]. The average response times for both the young and older groups of adults on each task was placed on a scatter-plot, and a regression line was then fitted to the data using the operation of y=mx+b. In doing so, a relationship was realized between the behavior in young adults and its ability to predict behavior on the same task in older adults. The power of this method of data analysis is that it can be used across task types to pool data from multiple studies. Here we harvest the powerful aspects of the Brinley analysis method and apply them to functional magnetic resonance imaging (fMRI) data. Meta-analyses of fMRI data are typically hindered by differences in the types of tasks used across studies. The use of the Brinley method allows for a meta-analysis of fMRI data that actually takes advantage of multiple cognitive tasks. However, the remaining challenge is to carefully interpret the findings.

The history of Brinley plots is rife with debate and discussion on what the plot is measuring and what it means. Feelings in opposing camps can even be so strong as to elicit T-shirts emblazoned with the No symbol over the word Brinley. Later researchers inferred that aspects of Brinley plots could provide information on general cognitive functioning in older adults since Brinley's data contained both task switching and nonswitching data subsets [2–7]. For example, if the slope for the fitted line of a group of tasks is 1, then there is equal change between tasks for younger and older adults. However, when the slope of the fitted line deviates and is greater than 1, there is an increased slowing in older adults associated with more cognitively demanding tasks (i.e., a general cognitive slowing deficit is evident in older adults regardless of task [e.g., [2]]). Other researchers have disagreed with this Brinley plot interpretation and suggest that the plot reflects a difference in response variability between the age groups rather than processing speed, per se [8]. Finally, current work in how aging affects processing speed questions the degree to which general cognitive slowing can be summarized with one linear function across all types of tasks [5, 9, 10].

In addition to describing the behavioral appearance of cognitive slowing, research has pursued localizing the phenomenon within the aging brain. Generalized cognitive slowing has been thought to result from pervasive slowing of all cognitive brain functions in older adults. With this in mind, some researchers have proposed that the locus of general cognitive slowing occurs where sensory impulses transfer to a common site of motor generation for the response and that this sensorimotor dysregulation intensifies with more difficult tasks [11–13]. Yordanova and colleagues found evidence to support this hypothesis using event-related potentials, which are able to evaluate the timing and strength of cognitive processing in response to external stimuli [11]. However, when considering task-specific slowing impact, other researchers point to additional slowing in specific cognitive areas such as working memory, visual search and mental rotation [10, 14], which may occur in addition to or instead of the sensorimotor slowing.

After reviewing the body of research literature on general cognitive slowing in aging, we adopted the early Brinley method for plotting reaction times and applied it to the blood-oxygen level dependent (BOLD) signal from multiple fMRI studies. In doing so, an attempt is made to localize brain areas responsible for the deviant slope in the response time Brinley plot. Unlike reaction times, the BOLD signal has a legitimate negative value (i.e., deactivations) that occurs when contrasting activity during two different events. For example, certain brain areas are more active during baseline than during any particular task. These areas prominently include posterior cingulate cortex and inferior parietal lobe areas and are known as the default network [15]. In applying Brinley's plotting method on the BOLD signal, we are able to construct novel Peiffer plots, a meta-analysis of fMRI data that is not limited to site locations of activation maxima and thus not skewed to evaluate only areas identified as statistically different within a study [see discussion in [16]]. Typical meta-analyses of fMRI data use location-centered approaches where the focus of peak activity is evaluated [16–20]. This can underestimate between-task differences because subthreshold activity differences are overlooked [see discussion in [16]]. Further, differences in task parameters and paradigm domain limit the tasks compared in many fMRI meta-analyses to a single type of task (e.g., Stroop interference task [16, 21]). With the novel method proposed here, a plot can be made across a variety of fMRI studies to evaluate two different population groups to identify areas showing between-task differences that may not necessarily be identified as deviant within an individual study comparison. Lastly, in using this method to compare young and older adults, we may be able to show localization of the areas that may in fact identify differences in age-related information processing that characterize general cognitive slowing.

## 2. METHODS

BOLD data and behavioral response times were obtained from 4 simple detection tasks (3 visual and 1 auditory). In order to plot a BOLD signal value for young and older adults in each study, original fMRI data was needed. The fMRI Data Center (http://www.fmridc.org) contributed a complete dataset from Buckner and colleagues (Accession no. 2-2000-1118W) for 2 points in the analysis (young = 14; older adults = 14) [22]. These BOLD signal measurements related to responses associated with the presentation of a single or double flashing checkerboard. The other 2 points were from studies performed in our laboratory (young = 20; older adults = 20) [23]. These BOLD signal measurements were related to block activity during an auditory task where subjects needed to respond when they heard a target tone or in a visual task where they responded to the blurring of a flashing checkerboard. For all points in the meta-analysis, all fMRI comparisons were between task and baseline (i.e., fixation cross) and were preprocessed with global signal correction. Further, during the preprocessing of the data, it is spatially normalized to MNI template space. Normalized task specific “con” images reflecting the task-related BOLD activity change from baseline were computed with SPM99 for all individuals in each dataset. These individual “con” images were then averaged within age group for each study resulting in a total of 8 average BOLD activity maps (2 age groups over 4 tasks). This process emulated the construction of a traditional Brinley plot which averaged the response times for each task within each age group. Within the BOLD average signal maps, each voxel contains a value representing the age group's average BOLD activity for that task at that standardized MNI x,y,z coordinate.

**Figure 1:**
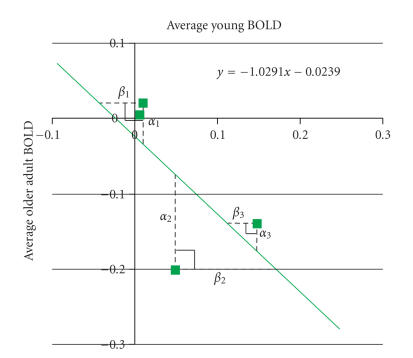
Representative voxel illustrating testing of the null hypothesis (H_0_ : m=1). To assess whether the null hypothesis is true in any given voxel, a t-test comparison of the α and β residual values was used. When α ≠ β, then the line was not significantly close to one and the voxel was considered to have a significant deviant slope. If the T value survived the correction for multiple comparison (3 contiguous voxels where FWE P < 0.05), the respective brain area was considered to be a loci for general cognitive slowing. A second analysis is needed to identify areas where voxels with slopes of −1 were eliminated in the residual analysis.

Using the 4 average young maps as observed x-values and the corresponding 4 average older adult maps as observed y-values, a linear regression analysis (y=mx+b) was calculated within each voxel that contained at least 3 x,y data points (see Figure 1 for a representative voxel). Individual 3D maps were computed that contained voxels with individual regression parameters of interest (e.g., slope, b-intercept, R-square, predicted y-value, etc.). Since the null hypothesis H_0_ in question was whether or not there was equivalent change between young and older adults across tasks, the slope value for H_0_ was 1. To evaluate H_0_ : m=1, the absolute residual values were calculated at each data point in SPM2 (α=|y−y^| where⁢  y^=mx+b⁢  and⁢  β=|x−x^| where⁢  x^=(y−b)/m). The resulting 8 residual maps (2 age groups over 4 tasks) were then statistically compared using a t-test in SPM. When the n is large enough, theoretically, it would be more statistically correct to analyze the difference between these residuals with a paired t-test. If the null hypothesis was true, then the absolute residual values would be equal and not statistically different from each other (α=β). If the slope was not equal to 1, then the voxel's t-test would be significant (α ≠ β). Multiple comparisons were controlled for by using FWE of P < 0.05 and an extent threshold of at least 3 consecutive voxels. The sign (+ or −) and value of the slope could then be assessed to determine how the two groups deviated in their BOLD signals across the tasks plotted in the analysis (e.g., one group activates an area more across tasks then the other group).

As graciously pointed out by an anonymous reviewer, slopes of −1 should not be considered as part of the null hypothesis, since the direction of activity across tasks for the age groups would actually be opposite (i.e., young adults activating across tasks while older adults deactivate). Therefore, it is also important to investigate areas where the slope is significantly negative, since the above “residual” analysis would not just eliminate slopes of +1 but also slopes which were not significantly different from −1. An example of this can be seen in the plotted data of Figure 1 in which this particular voxel would not be significant in the above “residual” analysis, but still represents an interesting result. Significant voxels with a slope of −1 can be identified using the p-value of the regression used to fit the Peiffer plot (i.e., P < .05 for the slope to be different from zero). Three contiguous voxels with a significant regression p-value and a negative slope will be considered a cluster of interest as well.

To evaluate a significant voxel's b-intercept, the 95% confidence interval was assessed. If this interval contained zero, the voxel's b-intercept was considered not to deviate from zero. Again, b-intercept clusters were considered significant if they contained at lest 3 contiguous voxels with significantly nonzero b-intercepts.

## 3. RESULTS

**Figure 2:**
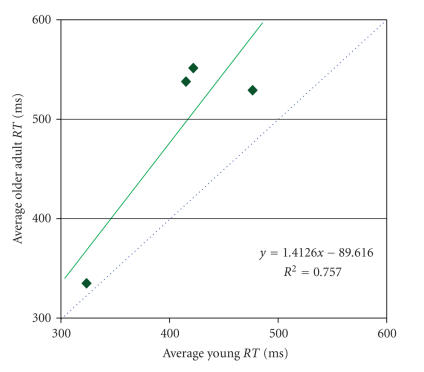
Traditional Brinley plot of response times for the 4 tasks used in the fMRI meta-analysis. If young and older adults showed equivalent between-task change in the speed of responses across these studies, the slope of the fitted line would be 1 (dotted blue line); however, results indicated that some general cognitive slowing is evident within the datasets since the slope of the fitted line was 1.4 (solid green line).

The traditional Brinley plot using average response times across the 4 tasks included in the fMRI meta-analysis showed the established differences between young and older adults (see Figure 2). The slope of 1.4 supports general cognitive slowing within the dataset even though relatively simple response time tasks were used, and although uninterpretable for response time data, the negative b-intercept is also typical. With a slope greater than one, older adults had greater differences between tasks in response time than younger adults.

**Figure 3:**
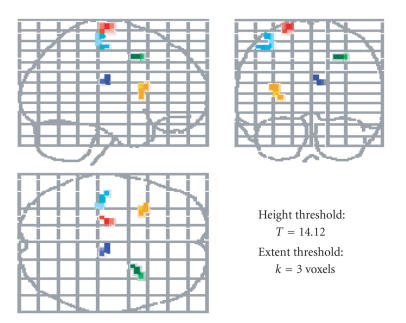
Contiguous clusters that survived FWE correction. These clusters had slopes that were not significantly equal to one (color-coded for clarity), and theoretically, they localized areas of differences in between-task BOLD signal change for older and younger adults.

**Figure 4:**
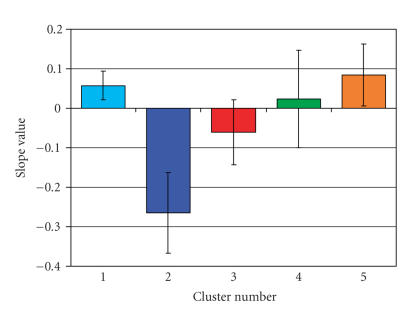
Average slope values for clusters with slopes significantly different from one. A total of 5 clusters (color-coded for clarity) survived and had average slope values less than 1 and greater than −1. These slope values are the result of greater between-task BOLD signal change in the cohort of young adults than older adults.

For the fMRI meta-analysis, five distinct clusters survived the stringent correction applied for multiple comparisons. The location of these clusters is summarized visually in Figure 3 and details are given in Table 1 . Clusters of interest to competing theories of general cognitive slowing were found within the left pre- and postcentral gyrus areas as well as within the right medial frontal gyrus. All clusters identified in the analysis had, on average, a slope that was significantly less than one yet significantly greater than negative one (see Figure 4). This slope indicates that between these 4 tasks, younger adults had greater BOLD signal change than older adults in these brain areas. Notably, there is a lack of difference in between-task BOLD activity within primary sensory areas such as vision between older and younger adults, even though several studies have reported older adults having less activity than younger adults in sensory areas [24–26].

**Figure 5:**
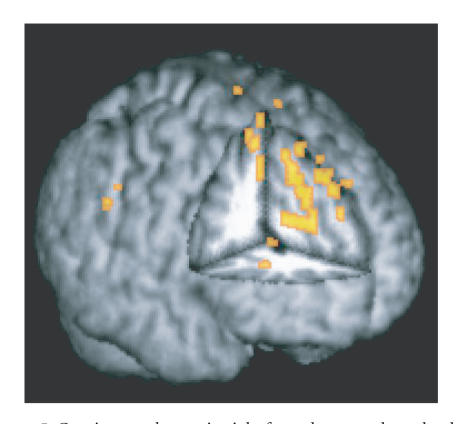
Contiguous clusters in right frontal cortex where the slope is −1. Several clusters were identified in the secondary analysis to assess for areas where activity was opposite in younger and older adults. These areas within right frontal cortex tended to be active across tasks in younger adults and deactivated across task in older adults. Further, these areas correspond to regions involved in attention and task decisions, which have also been implicated in general cognitive slowing theories.

When the Peiffer plot was explored for 3 contiguous voxels with significantly negative slopes less than zero, 19 total clusters were identified. Nine of these clusters (47%) were located within the right middle and superior frontal gyri and included a total of 68 voxels (see Figure 5). Across these clusters older adults showed BOLD deactivation on tasks when younger adults tended to slightly activate and older adults had BOLD activation when younger adults were deactivating on a task (average slope −1.89 +/− 0.21). These areas appear to be activating in opposition between the age groups and are contiguous to the right middle frontal gyrus area (cluster no. 4) identified in the “residual” analysis. Other clusters, showing similar activity differences were seen within left medial frontal gyrus (2 clusters; 6 voxels); left inferior parietal lobule (3 clusters; 10 voxels); cingulate gyrus (2 clusters; 7 voxels); and single clusters within the basal ganglia (5 voxels), midbrain (3 voxels), and the left posterior lobe of the cerebellum (7 voxels). It is important to note, however, that these findings, unlike those from the “residual” analysis above, have not been stringently controlled for multiple comparisons aside from retaining the requirement for 3 contiguous voxels.

To assess whether these slope findings were dependent on age and not an epiphenomenon of the datasets, a randomization of the age groups was performed within each dataset. Individuals were randomized in two groups so that the average age of both groups was roughly equal (*∼*51 years of age). When the Peiffer plot was constructed for these new groups, no significant clusters were identified where the null hypothesis (H_0_ : m=1) was false. Additonally, no significant areas were identified where the slope was −1. These findings thus support the claim that the results of the original plot were not due to the dataset composition (i.e., scanner, site, or paradigm) but were dependent on separating the study populations by age.

**Figure 6:**
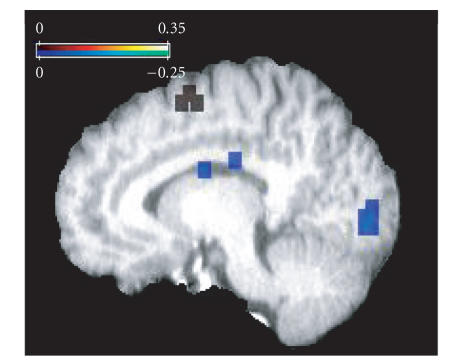
B-intercept map for Peiffer plot at x=10 mm. Cool-colored voxels show negative b-intercept values where older adults have lower activity than younger adults. Note the large cluster within the occipital area. Warm-colored voxels indicate positive b-intercepts where older adults show greater activity than younger adults. Interestingly, several contiguous voxels were identified within right motor cortex and indicated increased bilateral activity in older relative to younger adults across the 4 detection tasks.

Assessment of the b-intercept indicated that the lack of significant slopes within the primary sensory areas may be due to a baseline shift in activity between the age groups (see Figure 6). For example, within visual cortex, several areas were identified that had negative b-intercepts which indicated that across the tasks older adults tended to start from a lower BOLD activity level than young adults (if x=0, then y=a negative BOLD signal). This result is a continual within-task difference, which is also seen in the published literature [23–27]; however, since this reduced BOLD signal in older adults is constant across several tasks, it does not have a slope which deviates significantly from 1. In addition, an area within right motor cortex shows a positive b-intercept and thus greater activity in older adults relative to younger adults. As graciously pointed out by an anonymous reviewer, this result is consistent with the model hypothesis of hemispheric asymmetry in older adults (HAROLD). The HAROLD model states that there is reduced lateralization of brain activity in older adults relative to younger adults, which results from changes in neural architecture and not cognitive strategy [28, 29]. Due to the small number of data points used to construct these plots, the area included within the 95% confidence interval of the b-intercept is relatively large. Therefore, these early findings may underestimate the amount of brain activity which could be described as being affected by an age-related DC-shift and is thus an age-related BOLD signal difference that is independent of task.

Finally, an epiphenomenon of the method was revealed when evaluating the goodness of fit, as measured by the R-squared value. Areas where the slope was similar to one showed very high R-squared values (>0.8) suggesting a high predictability for older adults' BOLD signal in several brain regions; however, within the clusters identified as significantly deviant from one, R-squared values were lower and ranged between 0.1–0.46.

## 4. DISCUSSION

Here we report the preliminary use of a novel meta-analysis technique in studies on aging, which localizes one factor of general cognitive slowing to the sensorimotor transfer. These findings lend support to existing data from event-related potential work indicating that slowing occurs predominately during the time for generation of the response in older adults and not when evaluating incoming sensory material [11, 13]. Deviant slopes could be found in between-task BOLD activity values where younger adults have greater BOLD activity change than older adults in the sensorimotor transfer area in the left hemisphere. Additionally, right frontal areas were identified with slopes near −1, indicating that older and younger adults were activating these attentional areas in opposite directions across the tasks. Not surprisingly, more attentionally demanding tasks have shown that older adults have differential patterns of activation within the frontal cortex in response to the task when compared to younger participants [30, 31].

An important caveat exists, however, since datasets within this analysis were obtained from relatively noncognitively demanding tasks. In other words, more brain areas may be involved as loci of general cognitive slowing and would emerge as more cognitively demanding datasets become available for assessment. Comparing our current datasets to the existing literature on Brinley plots of reaction times, simple discrimination tasks show the least amount of response time slowing; therefore, these BOLD signal findings presumably will only become stronger with the addition of datasets containing more cognitively demanding conditions (e.g., working memory). In addition, with more tasks requiring greater attentional demand, the negative slope found within right frontal cortex may steepen and be found within the residual analysis which can control for the multiple comparisons inherent within imaging data. Power analyses of these preliminary results suggest that roughly 9 datasets are needed to perform a meta-analysis with a paired t-test to achieve a power-level of 80%.

Interestingly, all significant clusters identified as deviant within the meta-analysis were separate from peak activity differences reported in any of the individual tasks used within the datasets. If traditional meta-analysis techniques were used [16–20], none of these areas would have been found. Utilizing this novel meta-analysis technique, it is possible to assess between-task differences in BOLD activity between groups regardless of paradigm design, task parameters, and location of the scan. While global signal correction was used to normalize the datasets within this study, the assessment method could also be performed using average group z-maps that would allow datasets from multiple fMRI processing software packages to be analyzed collectively. Additionally, this method may yield interesting findings in a variety of study groups where a clear “normal” group can be identified and used on the x-axis (e.g., schizophrenics versus normals; dyslexics versus normals; AD versus normal older adults). It is important to keep in mind that the use of this method is to determine between-task differences among two populations and not to differentiate the two groups within any one paradigm of the analysis. Thus, differences in whether or not an area is identified as deviant come from how the BOLD signal responds across a wide array of tasks.

An existing disadvantage of this meta-analysis technique is that it requires access to raw fMRI data to obtain subjects' normalized contrast weighted BOLD activity maps (task - baseline) from multiple tasks. With the continued increase of complete data sets maintained in accessible repositories like the fMRI Data Center, this should hopefully become less burdensome in the near future.

Overall as a meta-analysis method in the fMRI field, this plotting addresses several limitations of existing analysis methods. Specifically, it allows the assessment of between-task differences regardless of a task's paradigm domain or baseline condition. Further, it can identify areas of subthreshold effects in addition to the suprathreshold within-task differences that are identified by performing a meta-analysis on voxel quadrants identified in individual studies as the local maxima. Lastly, this method provides imaging researchers the ability to localize between-task differences in BOLD signal and apply that knowledge to existing behavioral evidence not only in aging but in other complex conditions (e.g., dyslexia, schizophrenia, Alzheimer's disease, etc.) as well.

## Figures and Tables

**Table 1:** SPM volume summary

Cluster number	Talairach daemon label	Cluster size	FWE-corrected P value	T	x,y,z (mm)
1	Left post-central gyrus	7	.000	43.33	−44, −24, 55
2	Right thalamus	4	.000	41.35	8, −20, 20
3	Left pre-central gyrus	6	.000	37.85	−20, −20, 75
4	Sub-gyral right MFG	3	.001	27.48	28, 8, 45
5	Sub-gyral/Left Insula	5	.003	22.91	−36, 16, 15
